# Massive empyema caused by *Mycoplasma pneumoniae *in an adult: A case report

**DOI:** 10.1186/1471-2334-6-18

**Published:** 2006-02-01

**Authors:** Mony Shuvy, Moshe Rav-Acha, Uzi Izhar, Merav Ron, Ran Nir-Paz

**Affiliations:** 1 Department of Medicine, Hadassah-Hebrew University Medical Center, Jerusalem, Israel; 2 Department of Cardiothoracic surgery, Hadassah-Hebrew University Medical Center, Jerusalem, Israel; 3 Department of Clinical Microbiology and Infectious diseases, Hadassah-Hebrew University Medical Center, Jerusalem, Israel; 4 Department of Molecular & Cell Biology, 510 Barker Hall #3202, University of California, Berkeley, CA 94720-3202, USA

## Abstract

**Background:**

*Mycoplasma pneumoniae *is responsible for more than 20% of community acquired pneumonia cases, and capable of causing upper respiratory illness as well. Complications of *M.pneumoniae *infections include CNS involvement but other as pericarditis were also reported. The lack of feasible culture methods and under appreciation of the pathogens ability to cause invasive disease leads to reduced number of diagnosed *M.pneumoniae *related complications. In contrast to many other respiratory pathogens causing pneumonia, *M. pneumoniae *related severe pleural complications were almost never reported.

**Case presentation:**

We report a previously healthy 57 years old woman presented with indolent massive right pleural effusion, leukocytosis and elevated ESR. Extensive microbiological evaluation didn't reveal any pathogen in the pus even before antibiotic treatment was started. Surprisingly, *M.pneumoniae *DNA was detected in the pus from the empyema using PCR designed to detect *M.pneumoniae*. A serological assay (Serodia-Myco II) using convalescent serum was indeterminate with a titer of 1:80. The patient responded well to a treatment that included right thoracotomy with pleural decortication and a combination of antibiotics and anti-inflammatory medications.

**Conclusion:**

*M.pneumoniae *related empyema was never reported before in adult patients and was reported in only a few pediatric patients. In our patient there was no evidence to any common pathogens even before initiating antibiotic treatment. The only pathogen detected was *M.pneumoniae*. In this patient, serology was not helpful in establishing the diagnosis of *M.pneumoniae *related diseases, as was suggested before for older patients. We suggest that *M.pneumoniae *related empyema is probably under-diagnosed complication due to insensitivity of serology in older patients and under use of other diagnosis methods.

## Background

*Mycoplasma pneumoniae *is one of the most common respiratory pathogens [1]. Generally, it causes mild respiratory diseases managed by primary care physicians. The most common presentation is community acquired pneumonia in which it is responsible for more than 20% of cases, but is responsible as well for upper respiratory illness [1]. There are few very well documented complications caused by this pathogen. These mainly include CNS involvement but other complications as pericarditis are also reported [1]. There are a few reasons for the lack of evidence for *M.pneumoniae *involvement in complications of respiratory infections, such as: under appreciation of the pathogen as being able to cause invasive disease, and the lack of feasible culture methods as is the case with other pathogens [1,2]. Currently, diagnosis of *M.pneumoniae *infection [1-3] is based either on serology with it's limitations or PCR performed on clinical specimens – but the last method is not widely available in clinical microbiology labs. Here we report a case of massive empyema in adult patient caused by *M.pneumoniae *– a condition that was not reported before in this age group.

## Case presentation

A previously healthy 57 years old woman admitted to our hospital due to dyspnoea. Two weeks before admission she had right sided pleural pain followed by dyspnoea, without cough and without fever, these increased with time and therefore she referred herself to the hospital. Her physical examination suggested right sided pleural effusion, laboratory tests showed leukocytosis (WBC-19000/mm^3 ^with 89% neutrophils), HGB-12.5 g/dL, platelet count 400,000 platelets/mm3, and erythrocyte sedimentation rate 34 mm/hour. C-reactive protein level was highly elevated – 45 mg/dL (normal range 0–0.5 mg/dL). The results of chemistry panel were normal except for mildly elevated alkaline phosphatase – 157 u/l (40–130 u/l).

**Figure 1:**
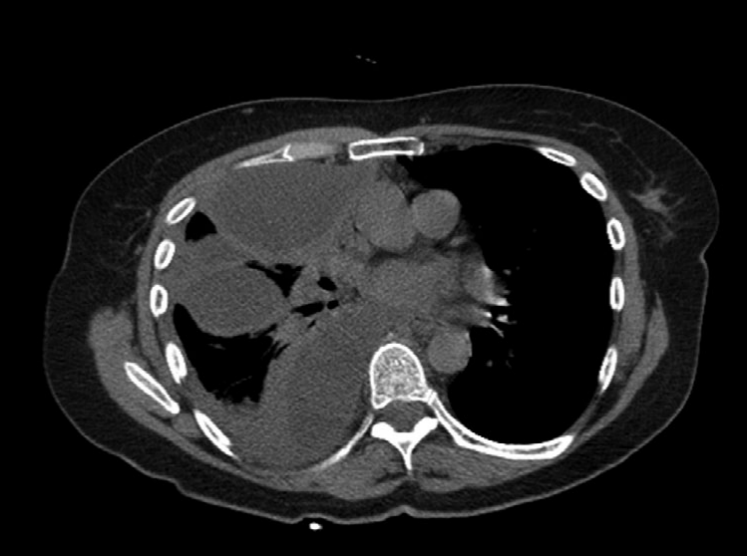
**Chest CT scan**. Chest CT scan of the patient showing massive right side pleural effusion with atelctasis of the lung.

A chest CT performed on admission (figure 1) showed massive right pleural effusion, with right lung athelectasis. Chest tube was inserted to the pleural cavity and evacuated only small amount of purulent fluid, although streptokinase was injected to the pleural cavity four times. Antibiotic treatment was started using IV Cefuroxime (750 mg three times a day) and oral Roxithromycin (150 mg twice a day) for 4 days without signs of improvement.

A repeated CT performed 4 days after admission, showed a massive amount of right sided pleural effusion with right middle and lower lobe athelectasis. Therefore, a right sided thoracotomy was performed. A significant amount of pus and fibrin was drained during the procedure, and the lung expanded normally after the pleura was decorticated on that side. Pus from the empyema (before and after starting antibiotic treatment) was cultured several times both for common pathogens as well as Mycobacteria but turned to be negative. Pathological examination of the pleura that was removed during the procedure showed fibrotic pleura covered by pus and fibrin without evidence for any organism. Given the clinical gradual and atypical course of the patient with the negative stains and cultures results, the pleural pus and fluid were also tested for *M.pneumoniae *using PCR. DNA was extracted from the pus using the DNAeasy tissue kit (Qiagen, Hilden, Germany). First we have utilised a Mycoplasma genus specific PCR amplifying 16SRNA DNA, doing direct amplification using the same thermal profile and GPO-3/MGSO primers as described in ref [4]. In this test one negative and one positive controls were used for 10 samples. Surprisingly a positive typical band at the size of ~270 bp was found, suggesting the presence of Mycoplasma *spp*. in the sample. Therefore, we further used two sets of *M.pneumoniae *specie specific PCR reactions to identify if it is *M.pneumoniae*. The first was a nested PCR using the primers of the ATPase gene in the same conditions as described in ref [5]. This test was shown in our lab to have a detection limit of ~20 CFU/ml (Nir-Paz *et al. *data on file). Two negative controls were included for every 8 samples tested in order to exclude contaminations during the nested reaction as well as a positive control for 16 reactions. This test gave a typical band at the size of 104 bp suggesting that the existence of *M.pneumoniae*. In order to confirm that finding, we have performed another *M.pneumoniae *specific PCR using the P1 gene. A direct PCR using the same primers and conditions as described in Ref [6] was performed. In this test one negative and one positive controls were used for 10 samples. Again this PCR gave a typical positive band at the size of 466 bp, confirming the presence of *M.pneumoniae *DNA in the sample. Unfortunately, only convalescent serum that was taken 30 days after the initial admission was available for serology, and it was indeterminate with a titer of 1:80 using the Serodia-Myco II assay (Fujirebio, Tokyo, Japan), and IgM negative using *Mycoplasma pneumoniaea *IgM EIA (ANILabsystems, Helsinki, Finland).

After the surgery, the patient was gradually recovered with intravenous azythromycin treatment (500 mg once a day) for 10 days followed by 5 more days of oral therapy (250 mg once a day), In addition she was treated with oral Pyranocarboxylate (400 mg twice a day) for the first two weeks after surgery.

The CRP level dropped to 9.5 mg/dl after the operation and two weeks later, the CRP level decreased into normal levels (0.2 mg/dl). At this time point the patient fully recovered without any breath or pain complaints and no residual fluid was seen in the chest X -ray.

## Discussion

We presented a case of *Mycoplasma pneumoniae *related massive pyogenic pleural effusion. There was no evidence of other causative agents in repeated stains, cultures or pathological examination of the pleura before and after the patient received antibiotics.

*M. pneumoniae *infections involves upper respiratory airway, lower respiratory airway or both; symptomatic disease typically develops gradually over a period of several days or weeks [1]. Bronchopneumonia involving one or more lobes develops in 3–10% of infected patients, while bilateral involvement occurs in about 20% of cases [1]. Minimal pleural effusion were suggested a few decades ago [7] in up to 20% of the patients when lateral decubitus films were performed, but diagnosis was based on cold agglutinin or complement fixation titers. Moreover, none of the patients in that study had any evidence of empyema. Few severe cases with significant pleural effusion and necrotizing pneumonia were reported in children [8]. Recently, two studies in these pediatric patients suggested that at least two distinct patterns of *M.pneumoniae *related pleural effusion [9,10]: the first is more benign, most probably reactive and does not contain *M.pneumoniae *DNA. The other, involves protracted disease and contains *M.pneumoniae *DNA. The pathogenesis of pleural effusion is still unclear, but the authors speculate that the presence of the DNA may elicit stronger immunologic reaction causing lung damage, with IL-8 and IL -18 play a role in the reaction [11,12]. In extensive literature search, no reports describing *M.pneumoniae *associated empyema in adult or elderly patients were found.

*M.pneumoniae *related empyema is currently considered a rare complication. Our case is unique by the old age of our patient and characterized by a subacute progress of a pulmonary disease. A massive empyema which requires thoracotomy and decortication, was never reported for this pathogen in this patient's age. Apparently, as was suggested before, serology in older people does not serve as a useful tool to support such diagnosis [13]. Nevertheless, it might be that *M.pneumoniae *is a common cause of empyema complicating respiratory infection and pneumonia but is under-diagnosed due to the use of insensitive diagnostic methods. We conclude that *M.pneumoniae *should be thought as an etiologic agent for respiratory infection followed by empyema in older patients. This should probably be confirmed by the use of PCR or by the use of other DNA based methods.

## Conclusion

In this case report we describe the first reported empyema caused by *Mycoplasma pneumoniae *in an adult. *M.pneumoniae *is one of the common respiratory pathogens, however due to it's fastidious nature it is not commonly detected. Therefore we suggest that *M.pneumoniae *related empyema is probably an under-diagnosed complication due to insensitivity of serology in older patients and under use of other diagnosis methods.

## Competing interests

The author(s) declare that they have no competing interests.

## Authors' contributions

MS, MRA, UI, and RNP cared for the patient and collected clinical data, MR performed the molecular diagnostic assays, MS and RNP drafted and revised the article. All authors read and approved the final manuscript.

## Pre-publication history

The pre-publication history for this paper can be accessed here:



## References

[B1] Waites KB, Talkington DF (2004). Mycoplasma pneumoniae and its role as a human pathogen. Clin Microbiol Rev.

[B2] Daxboeck F, Krause R, Wenisch C (2003). Laboratory diagnosis of Mycoplasma pneumoniae infection. Clin Microbiol Infect.

[B3] Loens K, Ursi D, Goossens H, Ieven M (2003). Molecular diagnosis of Mycoplasma pneumoniae respiratory tract infections. J Clin Microbiol.

[B4] van Kuppeveld FJ, van der Logt JT, Angulo AF, van Zoest MJ, Quint WG, Niesters HG, Galama JM, Melchers WJ (1992). Genus- and species-specific identification of mycoplasmas by 16S rRNA amplification. Appl Environ Microbiol.

[B5] Abele-Horn M, Busch U, Nitschko H, Jacobs E, Bax R, Pfaff F, Schaffer B, Heesemann J (1998). Molecular approaches to diagnosis of pulmonary diseases due to Mycoplasma pneumoniae. J Clin Microbiol.

[B6] de Barbeyrac B, Bernet-Poggi C, Febrer F, Renaudin H, Dupon M, Bebear C (1993). Detection of Mycoplasma pneumoniae and Mycoplasma genitalium in clinical samples by polymerase chain reaction. Clin Infect Dis.

[B7] Fine NL, Smith LR, Sheedy PF (1970). Frequency of pleural effusions in mycoplasma and viral pneumonias. N Engl J Med.

[B8] Narita M, Matsuzono Y, Itakura O, Yamada S, Togashi T (1998). Analysis of mycoplasmal pleural effusion by the polymerase chain reaction. Arch Dis Child.

[B9] Narita M, Tanaka H (2004). Two Distinct Patterns of Pleural Effusions Caused by Mycoplasma pneumoniae Infection. Pediatr Infect Dis J.

[B10] Wang RS, Wang SY, Hsieh KS, Chiou YH, Huang IF, Cheng MF, Chiou CC (2004). Necrotizing pneumonitis caused by Mycoplasma pneumoniae in pediatric patients: report of five cases and review of literature. Pediatr Infect Dis J.

[B11] Narita M, Tanaka H, Abe S, Yamada S, Kubota M, Togashi T (2000). Close association between pulmonary disease manifestation in Mycoplasma pneumoniae infection and enhanced local production of interleukin-18 in the lung, independent of gamma interferon. Clin Diagn Lab Immunol.

[B12] Tanaka H, Narita M, Teramoto S, Saikai T, Oashi K, Igarashi T, Abe S (2002). Role of interleukin-18 and T-helper type 1 cytokines in the development of Mycoplasma pneumoniae pneumonia in adults. Chest.

[B13] Daxboeck F, Kircher K, Krause R, Heinzl H, Wenisch C, Stanek G (2002). Effect of age on antibody titer to Mycoplasma pneumoniae. Scand J Infect Dis.

